# Draft Genome Sequence of *Diaporthe* sp. Strain HANT25, an Endophytic Fungus Producing Mycoepoxydiene

**DOI:** 10.1128/MRA.00805-20

**Published:** 2020-09-03

**Authors:** Kan Tulsook, Duangnate Isarangkul, Nongluksna Sriubolmas, Prasat Kittakoop, Suthep Wiyakrutta

**Affiliations:** a Department of Microbiology, Faculty of Science, Mahidol University, Bangkok, Thailand; b Department of Biochemistry and Microbiology, Faculty of Pharmaceutical Sciences, Chulalongkorn University, Bangkok, Thailand; c Chulabhorn Research Institute, Bangkok, Thailand; d Chulabhorn Graduate Institute, Program in Chemical Sciences, Chulabhorn Royal Academy, Bangkok, Thailand; e Center of Excellence on Environmental Health and Toxicology, Commission on Higher Education, Ministry of Education, Bangkok, Thailand; University of California, Riverside

## Abstract

*Diaporthe* sp. strain HANT25 is an endophytic fungus that produces mycoepoxydiene, a rare bioactive natural compound. Here, we report the genome sequence of *Diaporthe* sp. HANT25, comprising 55.3 Mb in 80 scaffolds. The genome sequence should enhance understanding of the biology and bioactive compound production potential of the genus *Diaporthe*.

## ANNOUNCEMENT

Fungi in the genus *Diaporthe* (and their *Phomopsis*-like state) are commonly found inhabiting a broad range of plant hosts as nonpathogenic endophytes, pathogens, or saprobes (1). They are capable of producing an array of structurally diverse low-molecular-weight bioactive secondary metabolites with antimicrobial, antiparasitic, or anticancer activities (2). *Diaporthe* sp. strain HANT25 (formerly known as *Phomopsis* sp. strain HANT25) is an endophytic fungus that was isolated from twigs of a Thai medicinal plant, Hydnocarpus anthelminthicus Pierre ex Laness, collected from Central Botanical Garden (Pukae), Saraburi Province, Thailand (3). The fungus was initially identified as a *Phomopsis* sp. based on both microscopic morphology and a molecular method using internal transcribed spacer (ITS) sequences (1). The ITS sequences were deposited in GenBank under the accession number EF635375. This endophytic fungus can produce mycoepoxydiene and derivatives that contain a rare oxygen-bridged cyclooctadiene structure and have cytotoxic activity toward cancer cell lines, as well as anti-inflammatory activities (3–6). The fungus was kept on agar slants under liquid paraffin.

The genomic DNA of *Diaporthe* sp. HANT25 was obtained from a 6-day-old culture of the fungus in 50 ml Sabouraud dextrose broth (Merck) at 25°C under static conditions. The mycelium was collected by filtration and then lyophilized. The genomic DNA extraction, library preparation, sequencing, and *de novo* assembly of the genome were performed by Macrogen (Republic of Korea). The genomic DNA was fragmented and a sequencing library was constructed with the TruSeq DNA PCR Free kit (Illumina, CA, USA) with 350-bp inserts. Whole-genome sequencing was performed using the Illumina HiSeq 2000 platform, generating paired-end reads of 100 bp. A total of 8,136,214,782 bases in 80,556,582 reads were obtained. Sequencing quality was inspected using FastQC (7), and the reads were filtered for pairs of paired-end reads that had >90% of bases with a base quality score of ≥Q20. The filtered reads, consisting of 6,528,808,872 bases in 64,641,672 reads, were *de novo* assembled using SOAPdenovo2 version 2.0.4-r240 (8) with a k-mer size of 25 estimated with Jellyfish version 1.1.11 (9). The genome sequence of *Diaporthe* sp. strain HANT25 consisted of 55,338,496 bases distributed among 80 scaffolds. The *N*_50_ value was 1,996,735 bases, and the GC content was 52.11%. The sequencing depth was estimated to be 89×. The genome assembly quality was evaluated using Benchmarking Universal Single-Copy Orthologs (BUSCO) (10) version 4.0.6 using genome mode with the Sordariomycetes_odb10 data set (as of 20 November 2019). Of 3,817 BUSCO genes, more than 98% were found in the genome assembly (complete, 98.6% [single, 98.3%; duplicated, 0.3%]; fragmented, 0.3%; missing, 1.1%). The availability of this genome should enhance understanding of the biology and the secondary metabolite production potential of the genus *Diaporthe*.

### Data availability.

The genome sequence of *Diaporthe* sp. strain HANT25 has been deposited in GenBank under the accession number JACBFG000000000 (version JACBFG010000000). The associated BioProject and BioSample accession numbers are PRJNA642004 and SAMN15375391, respectively. The raw sequence reads were deposited in the SRA under accession number SRR12145307.
